# Vitamin C for septic shock in previous randomized trials: implications of erroneous dosing, timing, and duration

**DOI:** 10.1186/s13054-022-03946-w

**Published:** 2022-03-15

**Authors:** Hyun Jung Lee, Ok-Hyeon Kim, Moon Seong Baek, Won-Young Kim

**Affiliations:** 1 Department of Anatomy and Cell Biology, Chung-Ang University College of Medicine, Seoul, Republic of Korea; 2 Department of Global Innovative Drugs, The Graduate School of Chung-Ang University, Chung-Ang University, Seoul, Republic of Korea; 3 Division of Pulmonary and Critical Care Medicine, Department of Internal Medicine, Chung-Ang University Hospital, Chung-Ang University College of Medicine, Seoul, Republic of Korea; 4 Biomedical Research Institute, Chung-Ang University Hospital, Seoul, Republic of Korea

**Dear Editor,**

We read with interest the article by Rosengrave et al. [1] on a pilot randomized trial to assess the efficacy of intravenous (IV) vitamin C for improving vasopressor requirements and other outcomes in patients with septic shock. They did not observe significant differences in the dose or duration of vasopressor use between the vitamin C and placebo groups. The authors argue that the study was limited by the small sample size (to detect differences in populations with a lower prevalence of vitamin C deficiency), insufficient dosage (median: 8 g/day), and delayed initiation of vitamin C (median: 18 h). Further studies considering these limitations are needed, although we would like to add several issues regarding vitamin C duration and patient heterogeneity.

Previous studies have demonstrated that some patients might experience hypovitaminosis C as early as 48 h after discontinuation of vitamin C infusion, regardless of the dosing regimen [2]. Given that most randomized trials that failed to show a survival benefit of vitamin C limited its use to a maximum of 4 days, sustained therapy may be needed to obtain the favorable effects of vitamin C over time. In the CITRIS-ALI trial that evaluated the benefit of IV vitamin C among patients with sepsis and acute respiratory distress syndrome, the mortality rate was 81% lower in the 4-day treatment group than in the placebo group [3]. However, there was no between-group difference in mortality after treatment cessation. In the study by Rosengrave et al. [1], the dosing regimen may have been “too short” to observe any treatment effects because half of the intervention group did not receive vitamin C for the entire 4 days. We recently assessed the efficacy of vitamin C, hydrocortisone, and thiamine in a murine model of sepsis induced by cecal ligation and puncture (CLP). Vitamin C (45 mg/kg), hydrocortisone (1.5 mg/kg), and thiamine (3 mg/kg) were dissolved in 1 mL lactated Ringer’s solution and administered intravenously starting 6 h after CLP and continued every 12 h for 4 days for a total of 8 doses. Fluid resuscitation and antibiotic therapy (25 mg/kg of imipenem in lactated Ringer’s solution with 5% dextrose starting 6 h after CLP and continued every 12 h for a total of 6 doses) were also provided. Notably, over 50% of control mice died within 3 days of CLP, whereas all treated mice survived the 4-day intervention and only started to die after cessation of intervention (Fig. 1a). Inflammatory biomarkers in serum measured 24 h after CLP (tumor necrosis factor-α) and in hepatocytes harvested 7 days after CLP (heme oxygenase 1 and prostaglandin-endoperoxide synthase 2) were significantly lower in the treated mice than in the control mice (Fig. 1b, c). In addition, hepatocyte swelling and necrosis 7 days after CLP were markedly attenuated in the treated mice (Fig. 1d). These findings are consistent with those in our previous nationwide cohort study that found significantly lower in-hospital mortality in sepsis patients treated with IV vitamin C for ≥ 5 days [4]. In this study, the survival curves of patients who received vitamin C for 1–2 or 3–4 days tended to diverge earlier during the course of sepsis than did those of patients treated for ≥ 5 days.

**Fig. 1:**
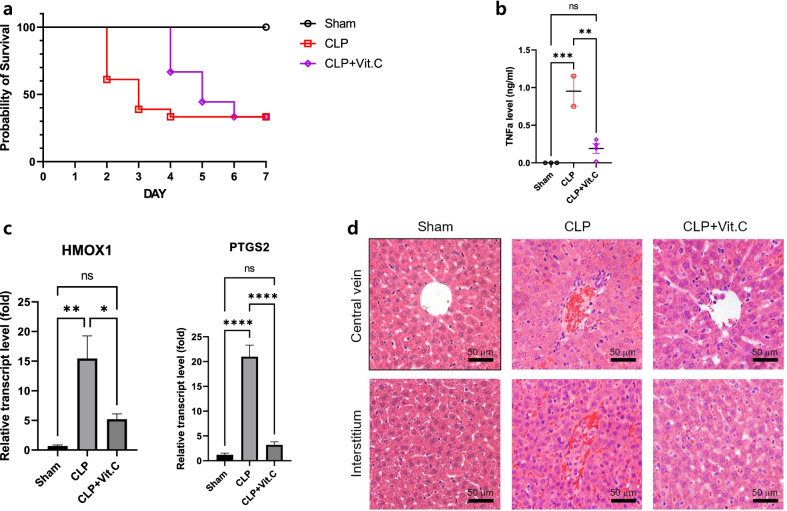
Vitamin C, hydrocortisone, and thiamine therapy in a pre-clinical sepsis model. **a** Among CLP mice, > 50% of those in the control group are dead within 3 days, whereas all mice in the intervention group are alive during the 4-day treatment period and only started to die after cessation of treatment. **b** After 24 h of CLP, serum TNF-α level (measured by ELISA) is significantly increased in CLP mice, whereas it is significantly reduced in treated mice. The data are shown as the mean ± SD. **p < 0.01 and ***p < 0.001; one-way ANOVA with Tukey’s multiple comparison test. **c** qRT-PCR quantification of HMOX1 and PTGS2 mRNA expression in hepatocytes during sepsis. After 7 days of CLP, relative quantification of HMOX1 and PTGS2 expression shows they are significantly increased in CLP mice, whereas they are significantly decreased in treated mice. The data are shown as a ratio of the treated mice to the controls (set as 1.0) and are presented as the mean ± SD. *p < 0.05, **p < 0.01, ***p < 0.001, and ****p < 0.0001; one-way ANOVA with Tukey’s multiple comparison test. **d** Liver tissues are harvested 7 days after CLP for histopathologic assessment. Representative liver sections showing reduced hepatocyte swelling and necrosis in treated mice (H & E staining; scale bar: 50 μm). *CLP* cecal ligation and puncture, *ELISA* enzyme-linked immunosorbent assay, *HMOX1* heme oxygenase 1, *ns* not significant, *PTGS2* prostaglandin-endoperoxide synthase 2, *qRT-PCR* quantitative real-time polymerase chain reaction, *TNF-α* tumor necrosis factor-α

Second, the outcomes of the previous trials may have been influenced by patient heterogeneity. Moreover, vitamin C may be more beneficial in certain sepsis subgroups, such as the “hyperinflammatory” subphenotype [5]. Older (age ≥ 70 years) patients with multiple comorbidities are more likely to be excluded from clinical trials evaluating agents for sepsis, often because of their risk of death irrespective of treatment response. In Rosengrave’s study [1], the median age of the participants was 69 years, and only 15% had comorbidities. However, our data demonstrated that these patients had lower mortality rates when vitamin C was administered [4]. Interestingly, there was no survival benefit in younger patients (< 70 years) and those with fewer comorbidities. One possible explanation for this discrepancy may be that older patients with comorbidities are more likely to have chronic vitamin C deficiency prior to sepsis, and thus, they are theoretically more likely to benefit from vitamin C treatment. Further studies are required to clarify these potential mechanisms.

In addition to the limitations described in the study by Rosengrave et al. [1], other limitations in previous randomized trials of vitamin C for septic shock include short-term vitamin C administration and heterogeneity of the populations. For patients with sepsis or coronavirus disease 2019, two randomized trials (LOVIT-COVID NCT04401150 and C-EASIE NCT04747795) are currently underway to assess the benefit of very high-dose (200 mg/kg/day) and early (within 6 h after admission) IV administration of vitamin C, respectively. However, their protocols also limit the use of vitamin C for 4 days, which may be unlikely to produce a profound impact on clinical outcomes.

## Data Availability

All data generated or analyzed during this study are included in this published article.

## Abbreviations

CLP: Cecal ligation and puncture
IV: Intravenous
